# Contraction of the Ventral Abdomen Potentiates Extracardiac Retrograde Hemolymph Propulsion in the Mosquito Hemocoel

**DOI:** 10.1371/journal.pone.0012943

**Published:** 2010-09-23

**Authors:** Jonathan W. Andereck, Jonas G. King, Julián F. Hillyer

**Affiliations:** Department of Biological Sciences and Institute for Global Health, Vanderbilt University, Nashville, Tennessee, United States of America; Universidade Federal do Rio de Janeiro, Brazil

## Abstract

**Background:**

Hemolymph circulation in mosquitoes is primarily controlled by the contractile action of a dorsal vessel that runs underneath the dorsal midline and is subdivided into a thoracic aorta and an abdominal heart. Wave-like peristaltic contractions of the heart alternate in propelling hemolymph in anterograde and retrograde directions, where it empties into the hemocoel at the terminal ends of the insect. During our analyses of hemolymph propulsion in *Anopheles gambiae*, we observed periodic ventral abdominal contractions and hypothesized that they promote extracardiac hemolymph circulation in the abdominal hemocoel.

**Methodology/Principal Findings:**

We devised methods to simultaneously analyze both heart and abdominal contractions, as well as to measure hemolymph flow in the abdominal hemocoel. Qualitative and quantitative analyses revealed that ventral abdominal contractions occur as series of bursts that propagate in the retrograde direction. Periods of ventral abdominal contraction begin only during periods of anterograde heart contraction and end immediately following a heartbeat directional reversal, suggesting that ventral abdominal contractions function to propel extracardiac hemolymph in the retrograde direction. To test this functional role, fluorescent microspheres were intrathoracically injected and their trajectory tracked throughout the hemocoel. Quantitative measurements of microsphere movement in extracardiac regions of the abdominal cavity showed that during periods of abdominal contractions hemolymph flows in dorsal and retrograde directions at a higher velocity and with greater acceleration than during periods of abdominal rest. Histochemical staining of the abdominal musculature then revealed that ventral abdominal contractions result from the contraction of intrasegmental lateral muscle fibers, intersegmental ventral muscle bands, and the ventral transverse muscles that form the ventral diaphragm.

**Conclusions/Significance:**

These data show that abdominal contractions potentiate extracardiac retrograde hemolymph propulsion in the abdominal hemocoel during periods of anterograde heart flow.

## Introduction

**Figure 1:**
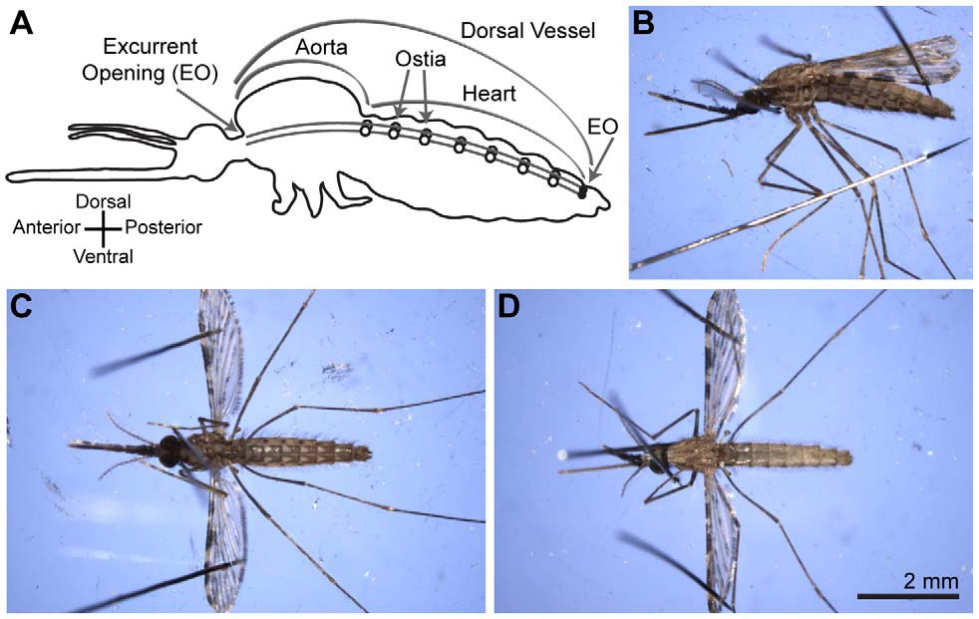
Mosquito body plan and positioning system. A. Mosquito body plan, illustrating the position of the dorsal vessel (divided into an abdominal heart and a thoracic aorta), the ostia (valves), and the excurrent openings. B–D. In this study, mosquitoes were restrained in lateral (B), ventral (C) and dorsal (D) orientations using non-invasive methods.

In insects, the transport of nutrients, wastes and hormones between cells, as well as effective immune surveillance and killing of foreign invaders in the hemocoel (body cavity), requires the continuous circulation of hemolymph (blood) throughout all regions of the insect [1], [2]. This circulation of hemolymph is controlled by a series of myogenic pumps, with the primary pump being a dorsal vessel that is located underneath the longitudinal midline of the dorsal cuticle and extends the length of the insect (Figure 1A).

In mosquitoes, the dorsal vessel is subdivided into a thoracic aorta and an abdominal heart [3]. The aorta lacks significant musculature and is not involved in hemolymph propulsion. Instead, hemolymph circulation in the thoracic and abdominal hemocoel is primarily controlled by the contractile action of the heart. The heart extends the length of the abdomen and is composed of striated muscle oriented in a helical twist with respect to the lumen of the vessel. Hemolymph enters the heart through unidirectional valves, called incurrent ostia, located as pairs at the thoraco-abdominal junction and the anterior portion of abdominal segments 2–7. Once in the heart, hemolymph is propelled by the peristaltic contraction of heart muscle, and it is the direction of these wave-like contractions that determines the direction in which hemolymph is propelled: contractions that originate in the posterior abdomen and propagate toward the head propel hemolymph in the anterograde direction while contractions that originate at the thoraco-abdominal junction and propagate toward the posterior abdomen propel hemolymph in the retrograde direction [3]. This process of heartbeat directional reversal is not restricted to mosquitoes (Diptera: Nematocera); it has also been observed in Lepidoptera, Orthoptera, Homoptera, Ephemeroptera, Neuroptera, Embioptera, Coleoptera, Hymenoptera and Diptera:Brachycera [4], [5], [6], [7], and is thought to function in maintaining the proper dissemination of molecules throughout the body, thermoregulation, the equilibration of hemolymph pressure, and tracheal ventilation. While the insect heart is considered myogenic, contraction dynamics are at least under partial neural control. For instance, the neurohormones crustacean cardioactive peptide (CCAP), FMRFamide and serotonin increase heart contraction rates in several insect orders [8], [9], [10], [11], and in *Drosophila melanogaster* CCAP and glutamate selectively regulate anterograde and retrograde heart rhythms, respectively [12], [13].

Hemolymph flow through the mosquito heart occurs at velocities greater than 8 mm/sec, signifying that molecules can cross the length of the body in less than one second [3]. However, once hemolymph is expelled from the dorsal vessel through excurrent openings located near the terminal ends of the insect, its velocity is dramatically reduced as it flows through the hemocoel prior to re-entering the heart through the ostia. Because of lower flow rates in the hemocoel, insects have developed a series of accessory pulsatile organs located at the base of the appendages to ensure continuous hemolymph flow to areas where hemolymph would otherwise not recirculate [14]. Moreover, because all hemolymph that enters the heart does so through ostia located in the dorsal-most portion of the abdomen, mechanisms must also exist to prevent hemolymph in the ventral abdomen from becoming stagnant.

While characterizing the structural and functional mechanics of the mosquito heart, we observed that the ventral abdomen periodically contracted. Because of the rhythmic periodicity of these contractions and the dynamic changes in hemolymph flow patterns that soon followed, we hypothesized that these contractions aid in recirculating hemolymph occupying the ventral portion of the abdomen. Here, we characterize the structural mechanics of these ventral abdominal contractions and show that they synchronize with anterograde heart contractions to potentiate retrograde hemolymph flow in the extracardiac abdominal hemocoel.

## Materials and Methods

### Mosquito rearing and maintenance

*Anopheles gambiae* (Sua2La hybrid) were reared and maintained in an environmental chamber at 27°C and 75% relative humidity with 12-hour light and 12-hour dark photoperiods, including 30-minute crepuscular periods at the beginning and end of each light cycle. Larvae were hatched in plastic pans and fed a slurry of fish food and baker's yeast daily. Following pupation, mosquitoes were transferred to 4.73 L containers with a fine-mesh marquisette top and, upon eclosion, adults were fed a 10% sucrose solution *ad libitum*. All experiments were carried out on 4-day-old adult female mosquitoes.

### Mosquito positioning and video recording

Mosquitoes were cold anesthetized (∼5°C) for approximately 60 sec before being restrained using 0.15 mm diameter minutien pins on polymerized Sylgard® 184 silicone elastomer (Dow Corning, Midland, MI). Mosquito restraint systems were carefully developed to restrict large-scale movements while being gentle in nature; the procedures are minimally invasive, as only pins placed through non-vascular regions of the wings penetrated insect tissue (Figure 1B–D). For the simultaneous visualization of hemolymph flow through the heart and ventral abdominal contractions, as well as the quantification of hemolymph flow speeds in the lateral abdomen, mosquitoes were restrained laterally by placing pins: (1) on either side of the cervical membrane such that they crossed dorsally and restrained the mosquito between the pins and the Sylgard; (2) through non-vascular portions of each wing after they had been teased away from the abdominal tergum; (3) between the wings and the anterior portion of the abdomen; (4) over the legs, entering the Sylgard posterior to the third leg pair; and (5) immediately dorsal of the longitudinal midline of the scutum (Figure 1B). For the measurement of hemolymph flow speeds in the ventral abdomen, mosquitoes were restrained ventral-side up by placing pins: (1) against (not through) the anterior pronotal lobe, and (2) through non-vascular areas (e.g. cell R3) of each wing without creating horizontal or vertical tension (Figure 1C). For the exclusive measurement of heart contraction dynamics, mosquitoes were restrained dorsal-side up using pin placement similar to that of mosquitoes restrained ventral-side up (Figure 1D) [3]. Once restrained, mosquitoes were allowed to equilibrate to 24°C and the necessary manipulations were performed (see below). Individual mosquitoes were imaged on a Nikon SMZ1500 stereo microscope (Nikon Corp., Tokyo, Japan) and 60-second video recordings were acquired using a CoolSNAP HQ^2^ digital camera (Photometrics, Tucson, AZ) connected to Nikon NIS-Elements Advanced Research software. Videos were captured using average rates of 43 frames s^−1^ (lateral positioning) and 27 frames s^−1^ (ventral positioning) and a calibrated gain of 4×.

### Simultaneous measurement of abdominal and heart contractions through the lateral abdomen

Three independent mosquito cohorts were used in these analyses. Each cohort consisted of 30 mosquitoes that originated from the same egg batch and emerged as adults on the same day. Once laterally restrained, approximately 50 nl of a solution consisting of 0.04% w/v 2 µm red fluorescent carboxylate-modified microspheres (excitation/emission maximum spectra of 580/605 nm; Invitrogen, Carlsbad, CA) in phosphate buffered saline (PBS; pH 7.0) was injected through the anepisternal cleft of the lateral mesothorax using a finely-pulled capillary needle with a terminal opening of 10–20 µm. After the particles disseminated with the hemolymph for 30–60 seconds, mosquitoes were epi-illuminated on a Nikon SMZ1500 stereo microscope using low-level fluorescence and low-level incandescent illumination, and video recordings were acquired.

For each mosquito, abdominal contractions were analyzed by manually annotating the start and stop times of each abdominal contraction period and the number of contractions within each period. Independently, the start and stop times of each anterograde and retrograde heart contraction period were annotated by visualizing the directional movement of fluorescent microspheres flowing through the heart. Once contractions of the ventral abdomen and heart were independently mapped, superimposing the abdominal and heart contraction time-stamped measurements from each individual mosquito correlated both sets of data. Videos of the first 10 mosquitoes from each cohort for which both abdominal contractions and heart contractions could be continuously visualized for the entire 60-second recording were used in the analysis. Reasons for mosquito exclusion included insufficient bead injections that limited heart analysis and violent movements by mosquitoes (e.g., kicking). Data from the three independent trials were statistically analyzed (see section 2.6), and after ANOVA determined that the three trials were statistically similar the data were merged and presented in the results section as a single dataset.

Measurements on abdominal contractions obtained by visualizing mosquitoes restrained in the lateral position included the number of ventral abdominal contractions per minute, the length of abdominal contraction periods, the abdominal contraction rate, and the percent time the abdomen spends contracting. The abdominal contraction rate represents the speed at which the abdomen contracts during periods of abdominal contractions, and was calculated by counting the number of abdominal contractions in a 60 sec recording and dividing this number by the amount of time the abdomen spent contracting. Abdominal contractions per period represents the number of abdominal contractions within a complete contraction period, and was calculated by dividing the number of contractions within complete contraction periods by the total number of complete contraction periods in that video. Here, an abdominal contraction period is defined as a series of abdominal contractions flanked by intervals of rest, and for calculations referring to complete contraction periods only those periods containing both start and end points were used in the analysis (periods that overlapped with the beginning and end of captured videos were discarded).

Measurements on heart contractions obtained by visualizing mosquitoes restrained in the lateral position included the percentage of time the heart contracts in the anterograde and retrograde directions, the frequency of heartbeat directional reversals (a switch from anterograde to retrograde contractions and vice versa), and the length of anterograde and retrograde contraction periods. For heart contractions, contraction periods are defined as intervals containing a series of consecutive unidirectional contractions that are flanked by heartbeat directional reversals.

Lastly, for the graphical representation of heart and abdominal contractions, videos were analyzed in ImageJ software (NIH). Briefly, videos were imported into ImageJ and an area where heart contractions could be clearly identified throughout the length of the video was selected, thresholded to define the area occupied by hemolymph during each contraction, and the area fraction change calculated using the “analyze particles” feature. In the output, area fraction peaks represent individual heart contractions. A similar procedure was then carried out to identify ventral abdominal contractions.

### Measurement of hemolymph flow in the lateral and ventral abdominal hemocoel

To determine hemolymph flow patterns in the abdomen, 2-µm-diameter fluorescent microspheres were intrathoracically injected into the hemocoel and allowed to mix with the hemolymph for 30–60 seconds. After major extracardiac hemolymph flow lines in the abdominal hemocoel were qualitatively identified, 60-second videos of the abdomens of 10 mosquitoes were acquired for each positioning system, and the trajectory of particles in major flow lines of the lateral and ventral abdomen were quantitatively tracked using the manual feature of the Object Tracking module of NIS-Elements. For each positioning system (lateral and ventral side-up), movement of 10 microspheres was tracked per mosquito: 5 microspheres were measured during periods of concurrent anterograde heart contractions and abdominal contractions, and 5 microspheres were measured during periods of anterograde heart contractions and abdominal rest. No data was collected during periods of retrograde heart contractions because abdominal hemolymph flow lines differ between periods of anterograde and retrograde heart contractions. Because microspheres are removed from circulation by sessile and circulating hemocytes [15], [16], [17], all particle tracking was done within the first three minutes following injection, and only particles for which we could detect fluid flow were tracked. Measurements obtained included distance traveled, distance from origin, velocity, velocity from origin, and acceleration. Velocity represents the gross distance an individual microsphere traveled (path length) over the amount of time it was tracked. Velocity from origin represents the net distance (shortest path or net displacement) a microsphere traveled over the amount of time it was tracked. Maximum acceleration represents the maximum value for microsphere acceleration over the entire interval for which it was tracked. The average microsphere was tracked for a path length of 416 µm.

### Measurement of heart contractions through the dorsal abdomen

For the exclusive measurement of heart contraction dynamics, mosquitoes were positioned dorsal-side up and imaged through the tergum. Measurements were made in a similar manner to when mosquitoes were imaged through the lateral abdomen, except that additional measurements were obtained. Measurements collected included percent time contracting in the anterograde and retrograde directions, the frequency of heartbeat directional reversals, the total contraction rate, the contraction rate when the heart contracts in the anterograde direction, and the contraction rate when the heart contracts in the retrograde direction.

### Statistical analyses

The data obtained from each cohort or trial was separated by treatment group and independently tested for normality using the Kolmogorov-Smirnov (KS) normality test. For comparing three or more groups, data deemed normal by the KS test were analyzed by one-way analysis of variance (ANOVA), and, if deemed significantly different, multiple comparisons were done using Tukey's test. If non-normal, comparison of three or more groups was done by the Kruskal-Wallis test. For the comparison of two groups of non-paired data, normal data were compared using the two-tailed unpaired Student's t-test. If non-normal, non-paired data were analyzed using the Mann-Whitney test. For the comparison of two groups of paired data, normal data were compared using the two-tailed paired Student's t-test. If non-normal, paired data were analyzed using the Wilcoxon matched pairs test. For all statistical tests, differences were deemed significant at P<0.05.

### Histochemical staining of the abdominal musculature

Fluorescence staining of abdominal muscle was performed as described [3]. Briefly, live mosquitoes were intrathoracically injected with 0.25 µl of a solution consisting of 1% Triton X (Thermo Fisher Scientific Inc., Waltham, MA), 0.3 µM phalloidin-Alexa Fluor 488 (Invitrogen), and 8% formaldehyde (Electron Microscopy Sciences, Hatfield, PA) in PBS and allowed to incubate for 15 min. For mosquitoes to be viewed as undissected whole mounts, a small cut was made in the terminal abdominal segment and specimens were rinsed by perfusion with PBS. Whole abdomens were mounted in glycerol and visualized from the exterior on glass depression slides. For mosquitoes to be viewed as dissected whole mounts, abdomens were dissected along a coronal plane to obtain the dorsal and ventral halves or along a sagittal plane to obtain the pleural halves, washed 3 times for 5 min each with 0.1% Tween 20 in PBS, and mounted on glass slides using Aqua-Poly/Mount (Polysciences Inc., Warrington, PA). Samples were imaged under fluorescence illumination using a Nikon 90i upright compound microscope connected to a Photometrics CoolSNAP HQ2 camera. Z-stacks were acquired using a linear encoded Z-motor, and all images within a stack were combined to form a focused image using the Extended Depth of Focus (EDF) module of NIS Elements.

## Results

### The ventral abdomen periodically contracts in the retrograde direction

Qualitative and quantitative observations of the lateral abdomen of 30 mosquitoes originating from 3 independent cohorts revealed that ventral abdominal contractions occur as series of consecutive contractions followed by periods of rest in which no contractions take place (Video S1). All ventral abdominal contractions initiate at the thoraco-abdominal junction and propagate in a wave-like peristaltic manner toward the posterior of the insect (retrograde direction), concluding in the last abdominal segment and resulting in ventrally directed abduction. The ventral abdomen contracts an average of 52.8 times per minute, but these contractions are aggregated within distinct contraction periods (consecutive contractions without rest) that last an average of 2.83 sec and contain 5.37 contractions each, indicating that when the abdomen contracts it does so at a rate of 1.98 Hz (Table 1). Altogether, abdominal contraction periods occupy 26.9 seconds of every minute, or 45% of the time.

**Table 1:** Quantitative analyses of ventral abdominal contractions.*

	*Abdomen* contraction direction			
	Anterograde		Retrograde	
	Average	S.D.	Average	S.D.
Contraction direction	0%		100%	
Contractions per minute	N/A		52.8	15.9
Contraction period length	N/A		2.83 sec	1.11
Contraction rate during contraction periods	N/A		1.98 Hz	0.34
% time contracting	N/A		44.75%	11.99

*n = 30; S.D. = standard deviation; N/A = not applicable.

### The ventral abdomen contracts during periods of anterograde heart contractions

To test whether a correlation exists between the contraction of the ventral abdomen and the contraction of the heart, video recordings of the lateral abdomen of mosquitoes were re-analyzed to annotate heart contractions. These analyses revealed that, between the three cohorts tested, the heart pumps hemolymph in the anterograde direction an average of 64% of the time and in the retrograde direction an average of 36% of the time (Table 2). The heart reverses direction an average of 19.2 times per minute and the lengths of anterograde and retrograde contraction periods average 3.96 sec and 2.30 sec, respectively. The data on the percentage of time the heart contracts in the anterograde and retrograde directions were consistent with our earlier analyses of the mosquito heart [3], although the average number of heartbeat reversals per minute is greater in this study, and hence, the average individual heart contraction periods are shorter.

**Table 2:** Quantitative analyses of heart and abdominal contractions.*

	*Heart* contraction direction			
	Anterograde		Retrograde	
	Average	S.D.	Average	S.D.
% time the heart contracts	63.64%	6.63	36.36%	6.63
Length of heart contraction periods	3.96 sec	0.91	2.30 sec	0.48
When abdominal contractions start	100%		0%	
When abdominal contractions end	94.53%	3.43	5.47%	3.43
% time into heart period when abdominal contractions start	20.73%	11.78	N/A	
% time into heart period when abdominal contractions end	93.25%	6.73	N/A	

*n = 30; S.D. = standard deviation; N/A = not applicable.

**Figure 2:**
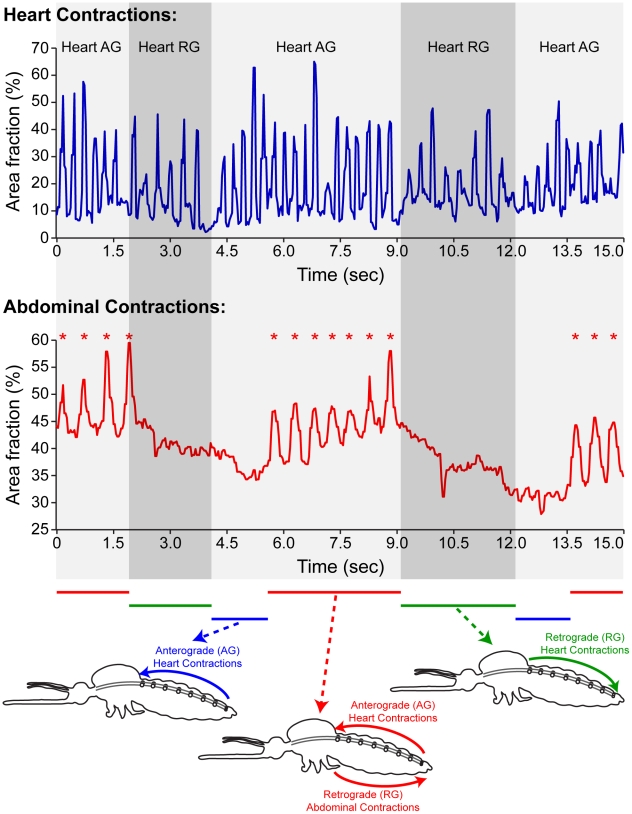
The ventral abdomen contracts in a retrograde direction during periods of anterograde heart contractions. Quantitative analysis of video S1 showing percent area changes in a section of the anterior heart (top graph) and a section of the posterior ventral abdomen (bottom graph) showing the temporal correlation of heart and abdominal contractions (peaks) during the three principal contraction periods: anterograde heart contractions and abdominal rest, anterograde heart contractions and ventral abdominal contractions, and retrograde heart contractions and abdominal rest (bottom diagram). Overall, ventral abdominal contractions (asterisks) correlate with anterograde heart contractions. Shaded boxes depict the direction of heart contraction periods (AG, anterograde; RG, retrograde).

Concurrent analysis of heart and ventral abdominal contractions revealed that 100% of abdominal contractions originate when the heart is contracting in the anterograde direction (Table 2; Figure 2). Of these abdominal contractions, 95% propagate exclusively during periods of anterograde heart contractions. The remaining 5% of abdominal contractions originate while the heart contracts in the anterograde direction but conclude after a heartbeat directional reversal.

Lastly, to ensure that the mosquito restraint system used in this study was not by itself inducing the observed abdominal contractions, we qualitatively visualized the heart and abdomen of unrestrained mosquitoes that (1) had their wings and legs removed or (2) had been cold anesthetized. In all cases, ventral abdominal contractions were readily apparent and correlated with anterograde heart periods (data not shown). Taken altogether, the above observations illustrate that retrograde ventral abdominal contractions occur in concert with anterograde heart contractions (Video S1; Figure 2).

### Ventral abdominal contraction periods begin approximately 20% into anterograde heart contraction periods and end upon a heartbeat directional reversal

The ventral abdomen contracts for an average of 26.9 seconds per minute (45% of the time). This number is significantly lower than the amount of time the heart spends contracting in the anterograde direction (38.2 sec/min, or 64% of the time), so despite the finding that the abdomen contracts almost exclusively as the heart pumps in the anterograde direction, it only does so an average of 70% of that time. Analysis of the timing of the beginning and end of ventral abdominal contraction periods revealed that abdominal contractions begin an average of 0.79 sec into anterograde heart contraction periods (or 21% into the period; Table 2) and end within 0.25 sec of a heartbeat reversal (or 93% into the period). When analyzed in the context of individual contractions, the last ventral abdominal contraction usually ended during the anterograde heart contraction immediately preceding a heartbeat reversal (Figure 2).

### Abdominal contractions potentiate retrograde extracardiac hemolymph flow in the abdominal hemocoel

Because there is a strong correlation between abdominal and heart contractions, we tested whether abdominal contractions play a direct role in altering hemolymph flow in the abdominal hemocoel. We have previously shown that during anterograde heart contractions hemolymph is expelled from the dorsal vessel through an excurrent opening located near the head (Figure 1A) [3]. Similarly, during retrograde heart contractions hemolymph is expelled from the dorsal vessel through an excurrent opening located in the 8^th^ abdominal segment (Figure 1A) [3]. Here, we intrathoracically injected fluorescently labeled 2 µm diameter microspheres and tracked their flow in the lateral and ventral abdominal hemocoel during the three principal contraction periods: (1) periods of retrograde heart contractions and abdominal rest, (2) periods of anterograde heart contractions and abdominal rest, and (3) periods of anterograde heart contractions with simultaneous ventral abdominal contractions (Figure 2).

Observation of microsphere movement in the lateral and ventral portions of the abdomen revealed that flow in the abdominal cavity is variable due to the lack of distinct hemolymph vessels. However, in general, the periodic discharge of hemolymph from the heart and into the posterior abdomen during periods of retrograde heart contractions results in net anterograde hemolymph flow in the extracardiac abdominal hemocoel (Videos S2 and S3). This anterograde flow occurs in discrete pulses that reflect the bolus-like discharge of hemolymph from the dorsal vessel that is associated with each retrograde heart contraction.

**Figure 3:**
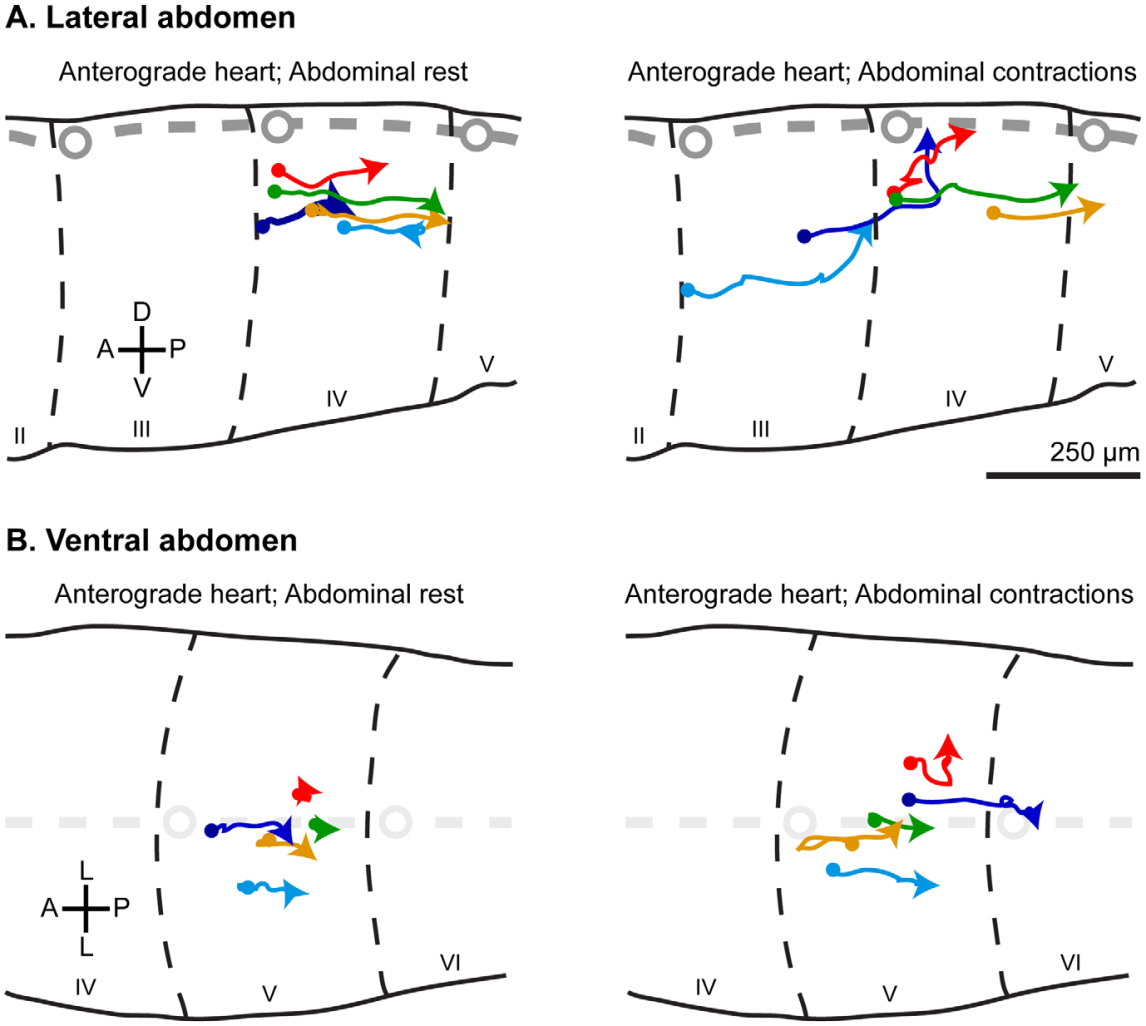
Hemolymph flow in the lateral and ventral mosquito abdomen. Intrathoracically-injected fluorescent microspheres were tracked as they flowed in the lateral (A) and ventral (B) abdomen during periods of anterograde heart contractions and abdominal rest, and during periods of anterograde heart contractions and ventral abdominal contractions (0.55 seconds of tracking data are shown per microsphere). In both the lateral and ventral abdomen, microspheres flowed in retrograde and dorsal directions, and achieved higher net displacement during periods of ventral abdominal contractions. Roman numerals denote abdominal segment number. D, dorsal; V, ventral; A, anterior; P, posterior; L, lateral.

Observations during periods of anterograde heart contractions revealed that extracardiac hemolymph entering the lateral abdomen after being expelled through the anterior excurrent opening of the aorta flows primarily in retrograde and dorsal (toward the ostia) directions, but with periods of random migration with no net positive displacement (Figure 3, Video S2). When the abdomen begins to contract, the directional movement of extracardiac hemolymph remains in a dorsoretrograde direction, but the periodic oscillations are more pronounced, and the net positive displacement occurs at a faster rate (Figure 3, Video S2). In the ventral abdomen, flow also occurs in a similar manner, but with less pronounced dorsoretrograde flow (Figure 3, Video S3).

**Figure 4:**
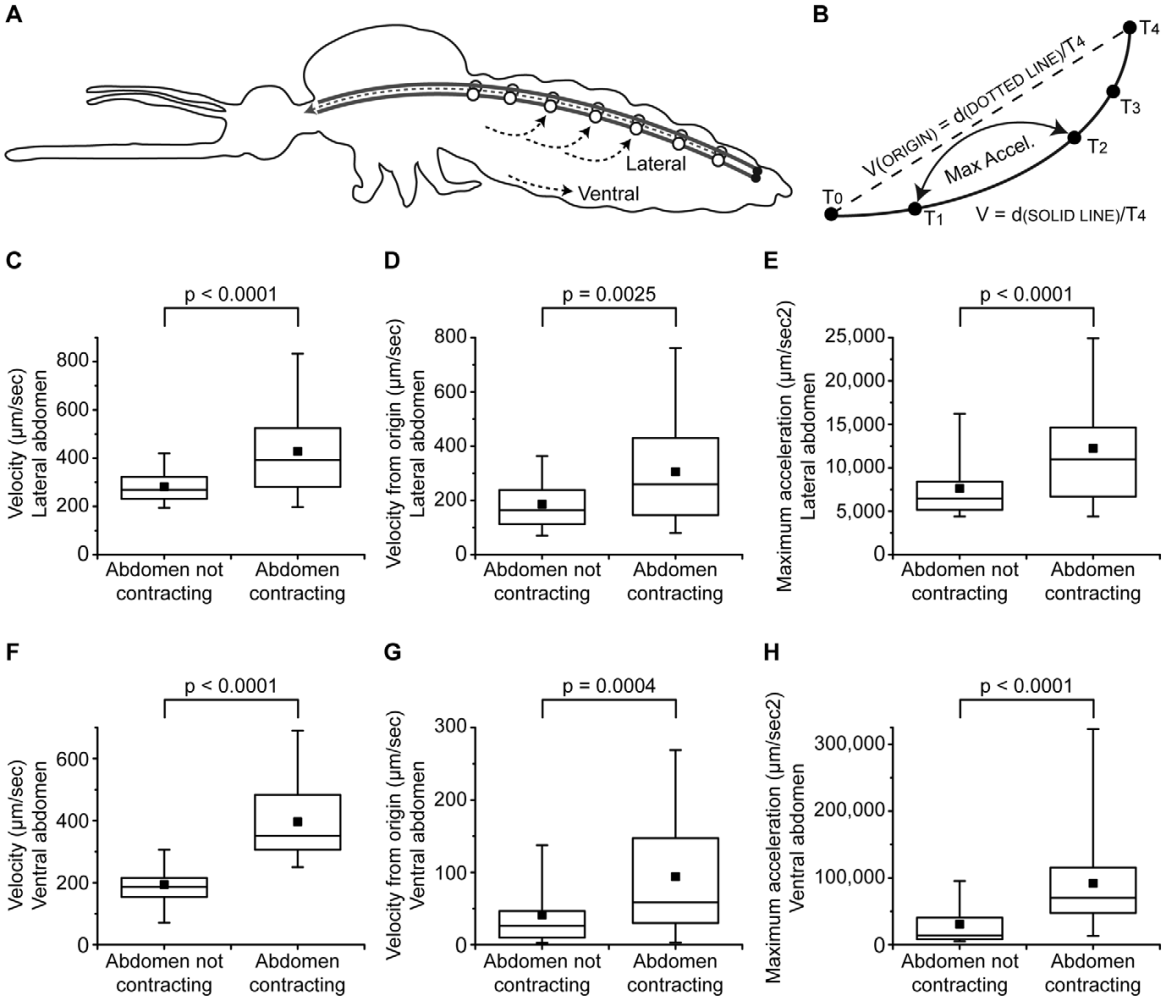
Hemolymph flow velocity and acceleration in the lateral and ventral mosquito abdomen. A. Diagrammatic representation of the general path of microspheres in the lateral and ventral abdomen during periods of anterograde heart contractions (black dotted arrows), illustrating the location where microspheres were tracked for velocity and acceleration calculations (panels C–H). B. In panels C–H, velocity represents the actual distance traveled (gross displacement; d, solid line) divided by time (T), velocity from origin represents the net displacement (d, dotted line) divided by time (T), and maximum acceleration represents the highest acceleration measured for a given microsphere. C–H. Velocity (C, F), velocity from origin (D, G), and maximum acceleration (E, H) of 2 µm fluorescent microspheres flowing through major lateral (C–E) and ventral (F–H) hemolymph flow lines. During periods of anterograde heart flow, hemolymph flows through the lateral and ventral abdomen at higher velocities and greater acceleration during periods of ventral abdominal contractions as compared to periods of abdominal rest (Mann-Whitney). Median, center line; 50% of the data, box; 90% of the data, whiskers; mean, solid square.

To quantify changes in extracardiac abdominal hemolymph flow during periods of anterograde heart contractions and the presence or absence of abdominal contractions we quantitatively tracked the trajectory of microspheres as they flowed along the lateral and ventral abdomen (Figure 3–4). During periods of abdominal rest, hemolymph flows through the lateral abdomen at a median velocity of 269 µm/sec with a median maximum acceleration of 6,471 µm/sec^2^. In contrast, during periods of abdominal contractions, hemolymph flows through the lateral abdomen at a median velocity of 392 µm/sec with a median maximum acceleration of 10,980 µm/sec^2^. Similarly, hemolymph velocity in the ventral abdomen changes from 186 µm/sec during periods of abdominal rest to 351 µm/sec during periods of abdominal contractions. Hemolymph acceleration also increases with the onset of abdominal contractions: the median maximum acceleration rises from 14,047 µm/sec^2^ during periods of abdominal rest to 70,386 µm/sec^2^ during periods of ventral abdominal contractions. Lastly, when hemolymph velocity was measured such that it only accounts for net displacement (rather than actual/gross displacement; Figure 4B) the same trend is seen: abdominal contractions increase the median velocity from origin from 164 µm/sec to 259 µm/sec and from 26 µm/sec to 59 µm/sec in the lateral and ventral abdomen, respectively. Hence, in both the lateral and ventral abdomen hemolymph flows faster (Mann-Whitney velocity p<0.0001 and velocity from origin p≤0.0025) and accelerates more violently (Mann-Whitney p<0.0001) during periods of ventral abdominal contractions. Lastly, while hemolymph flow is slower in the ventral abdomen when compared to the lateral abdomen, the effect of abdominal contractions on changes in hemolymph velocity, velocity from origin, and maximum acceleration is 29%, 43% and 295% greater, respectively, in this region of the insect. The more pronounced role of abdominal contractions in propelling hemolymph in the ventral abdomen was expected given that hemolymph in this area flows adjacent to the musculature responsible for ventral abdominal contractions.

### The ventral diaphragm, the lateral intrasegmental muscles, and the ventral intersegmental muscles are the primary drivers of ventral abdominal contractions

**Figure 5:**
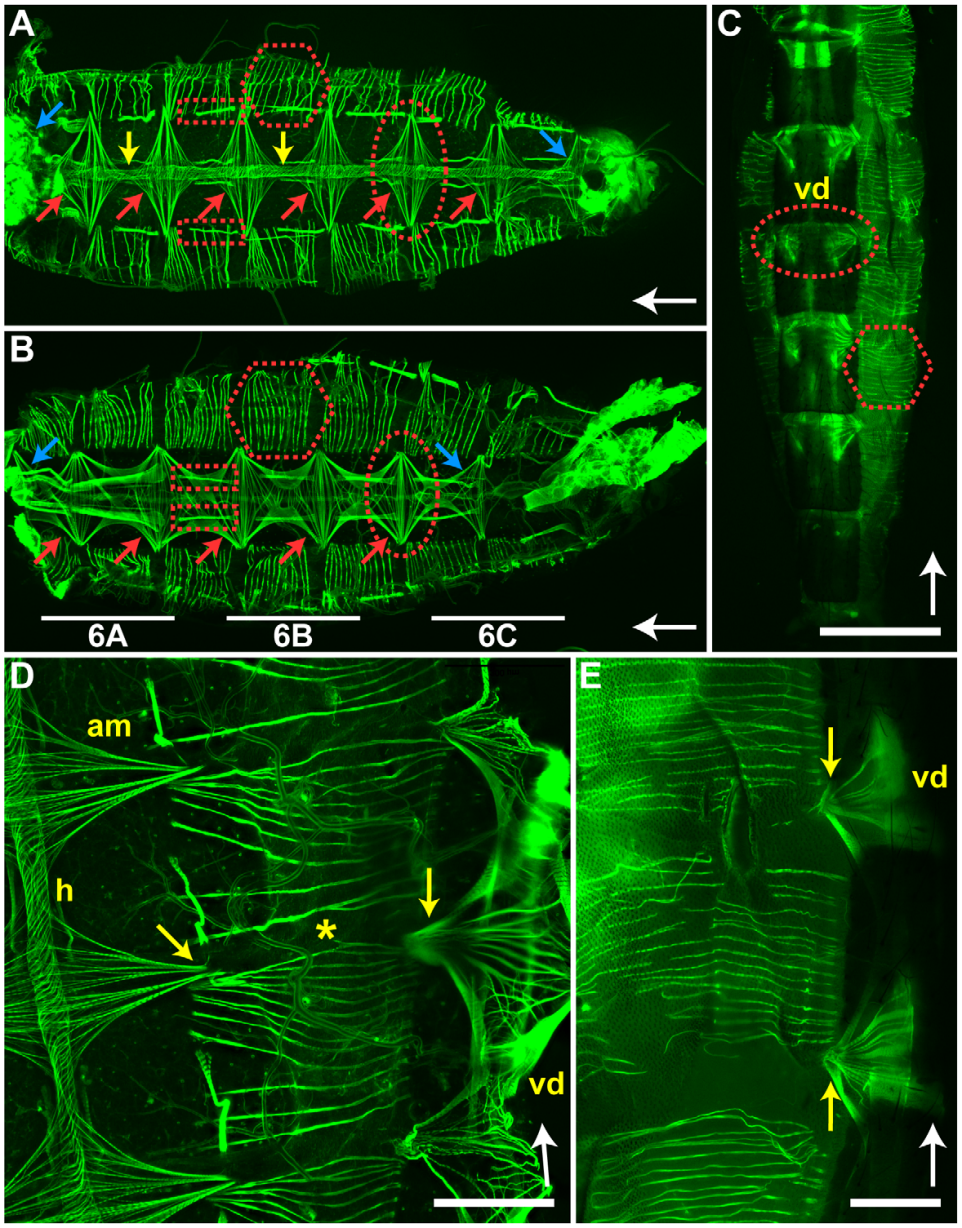
Abdominal musculature. A. Dissected whole mount showing that the abdominal musculature in the dorsal tergum is composed of (1) six complete pairs (red arrows) and two incomplete pairs (blue arrows) of alary muscle pairs (e.g., dotted circle) that support a muscular heart (yellow arrows), (2) paired intersegmental muscle bands (e.g., dotted rectangles) that connect adjoining tergites, and (3) intrasegmental lateral muscle fibers (e.g., hexagon) that extend from the lateral tergum to the lateral sternum. B. Dissected whole mount showing that the abdominal musculature in the ventral sternum is composed of (1) five complete pairs (red arrows) and two incomplete pairs (blue arrows) of ventral transverse body muscles (e.g., dotted circle) that laterally extend across sternites and form the ventral diaphragm, (2) paired intersegmental muscle bands (e.g., dotted rectangles) that connect adjoining sternites, and (3) intrasegmental lateral muscle fibers (e.g., hexagon) that extend from the lateral sternum to the lateral tergum. The underlined areas of this specimen, or an analogous specimen, are magnified in figure 6A–C. C. Whole mount imaging through the sternum showing the ventral diaphragm (vd; one ventral transverse muscle pair is circled) and the lateral intrasegmental muscles (e.g., hexagon). D. Whole mount of a specimen dissected along a sagittal plane showing the heart (h), three alary muscle pairs (am), three ventral transverse body muscle pairs (vd), intersegmental muscles, and intrasegmental lateral muscles (asterisk). Note that the alary and transverse muscles are attached to the lateral edges of the tergum and sternum (yellow arrows), respectively, and overlap with the intrasegmental lateral muscles that span the pleuron. E. Whole mount imaging through the pleuron and sternum showing the origin of ventral transverse muscle pairs (yellow arrows). Bars: A–C = 500 µm; D = 200 µm; E = 100 µm. White arrows point toward the anterior of the mosquito.

**Figure 6:**
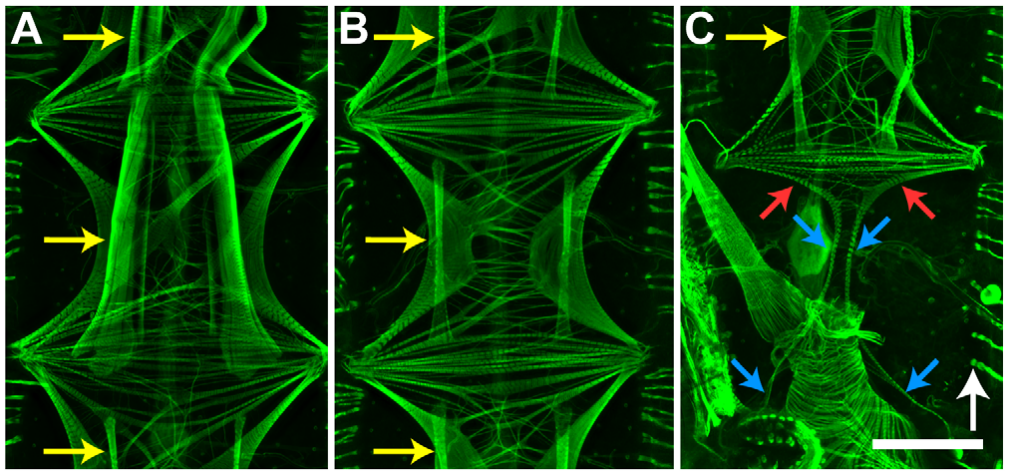
Ventral diaphragm. A–C. Imaging of anterior (A), middle (B), and posterior (C) segments of the ventral diaphragm. Note that the intersegmental muscle bands (e.g., yellow arrows) are considerably more robust in abdominal segments 1 and 2 (A). Furthermore, the terminal incomplete transverse body muscle pair is located in the anterior portion of abdominal segment 7 (C; red arrows), but extends bilaterally symmetrical myofibers that anchor on the 8^th^ sternite (C; blue arrows). Bar = 150 µm. White arrow points toward the anterior of the mosquito.

Given the importance of ventral abdominal contractions in potentiating extracardiac retrograde hemolymph flow in the abdominal hemocoel, we performed histochemical analyses to uncover the musculature driving these contractions. Staining of f-actin using Alexa Fluor-conjugated phalloidin revealed that the abdominal wall contains musculature in four distinct orientations: alary muscles, intrasegmental lateral muscles, intersegmental muscle bands, and transverse body muscles (Figures 5, 6).

The dorsal tergum contains 6 complete pairs (abdominal segments 2–7) and 2 incomplete pairs (abdominal segments 1 and 8) of alary muscles that aid in the opening and closing of the ostia [3]. Alary muscles originate as myofiber bundles that are anchored to distinct points in the anterolateral region of the tergites. Myofibers fan out and bind the heart (giving them their aliform shape), after which point they either join myofibers originating from the paired alary muscle on the other side of the heart or myofibers originating from an alary muscle in the adjoining abdominal segment.

The ventral sternum contains 5 complete pairs (abdominal segments 2–6) and 2 incomplete pairs (abdominal segments 1 and 7) of transverse body muscles that collectively form the ventral diaphragm and abduct the abdomen in a ventral orientation. Myofibers originate from distinct points in the anterolateral region of the sternites and extend toward the ventral midline, where they join myofibers from the paired transverse muscle originating on the other side of the ventral nerve cord or myofibers originating from a transverse muscle in the adjoining abdominal segment. These muscles are distinct from the dorsal alary muscles in that they are more robust, and emphasize the connection between abdominal segments rather than the connection across an abdominal segment.

Besides the musculature associated with the dorsal and ventral diaphragms, intrasegmental lateral muscle fibers span the pleuron and dorsoventrally compress the abdomen by anchoring in the lateral edges of the tergum and sternum. Greater than 10 distinct lateral myofibers originate in each abdominal segment, all of which travel parallel to one another along the cuticular wall.

Lastly, bilaterally symmetrical pairs of intersegmental dorsal and ventral muscle bands span the abdominal sutures and compress adjacent tergites and adjacent sternites, respectively. In the tergum, intersegmental muscle bands anchor to the lateral edge of the tergites (in a position immediately anterior of where the alary muscles join the tergites) and extend to the midway point of the adjoining anterior tergite. In the sternum, intersegmental muscle bands extend in a similar manner, except that they are located underneath the ventral diaphragm and in closer proximity to the ventral midline.

Extracardiac hemolymph propulsion in the abdomen occurs through the periodic contraction of muscle fibers spread throughout the abdominal wall. Intrasegmental muscle fibers that join the tergites to the pleurites dorsoventrally compress the abdomen. Intersegmental ventral muscle bands compress adjacent sternites and, in concert with the contraction of the ventral transverse body muscles, ventrally flex the abdomen.

### Mosquito manipulation has a negligible effect on heart contraction dynamics

In the current study we developed novel methods for the measurement of heart and abdominal contractions as well as extracardiac hemolymph flow. Because the potential impact of these methods on the biology of mosquitoes had not been measured, we carried out a series of trials that compared heart contraction dynamics following different manipulations. Data were analyzed in two ways. First, comparison of paired data from individual mosquitoes before and after a manipulation revealed whether that manipulation affected heart contraction dynamics. Second, comparison of data from the control groups of independent mosquito cohorts revealed the natural variation in contraction dynamics.

Analyses of paired data revealed that 4 of the 5 manipulations tested (positioning, incandescent illumination, injection, and recovery) had no effect on any of the six factors tested (% time contracting in the anterograde and retrograde directions, heartbeat reversals per minute, and total, anterograde and retrograde contraction rates; Figure S1). The other manipulation, fluorescence illumination, affected 5 of the 6 factors tested (Figure S1). Moreover, comparison of the control groups of the independent mosquito cohorts revealed no statistical difference for any of the six factors measured (Figure S1), but analysis of the cohort averages did detect considerable variation within the data: the ratio of the maximum vs. minimum averages of the control groups for the six factors measured (% time contracting in the anterograde and retrograde directions, heartbeat reversals per minute, and total, anterograde and retrograde contraction rates) were 1.17, 1.44, 1.92, 1.16, 1.14 and 1.14, respectively. Interestingly, similar analysis of the fluorescence treatment group (experimental vs. control) yielded ratios of 1.05, 1.16, 1.21, 1.16, 1.14 and 1.26, indicating that the difference between the averages is smaller for the fluorescence trial (with the exception of the retrograde contraction rate) than for the independent control cohorts. Taken altogether, these analyses indicate that there is considerable inherent biological variation within this physiological system, and confirms the appropriateness of the methods used in this study.

## Discussion

The data presented herein unequivocally show that ventral abdominal contractions are an integral component of the mosquito circulatory system. Specifically, the abdomen contracts in a retrograde peristaltic manner exclusively during anterograde heart contraction periods, resulting in increased extracardiac hemolymph propulsion in a dorsoretrograde direction. The data were remarkably consistent across the three cohorts tested, and control trials showed that the methods used to measure heart and abdominal contraction activity did not significantly affect normal physiological functions. The strong, exclusive correlation between abdominal and anterograde heart contractions suggests that abdominal contractions play more than an accidental role in insect hemolymph propulsion and are essential for proper hemolymph circulation.

Abdominal contractions have been described in several insect orders, including Coleoptera, Diptera, Hymenoptera, Lepidoptera, Blattodea and Orthoptera [5], [18], [19], [20], [21], [22], [23]. These contractions range from the barely discernible to the very pronounced, and some insects display more than one distinct type of abdominal contraction [18], [22], [23]. While the precise function of abdominal contractions continues to be debated, it has repeatedly been shown that they alter hemolymph pressure in the hemocoel [20], [24], [25], [26], and thus, have been hypothesized to function in hemolymph circulation, thermoregulation, development, and active ventilation [23], [27], [28], [29], [30].

To date, only indirect evidence has linked abdominal contractions and hemolymph propulsion. The majority of studies on this subject have inferred a circulatory role after measuring hemolymph pressure changes associated with abdominal contractions using invasive techniques that physically inserted probes into the hemocoel or techniques that detected outer cuticular movements [22], [23], [26]. Additional indirect evidence supporting the connection between abdominal contractions and hemolymph circulation includes the finding that allatropin and serotonin modulate both abdominal and heart contractions in the moth, *Pseudaletia unipuncta* [19]. In the present study we report a conclusive link between abdominal contractions and heart contractions and for the first time show that ventral abdominal contractions increase the rate of extracardiac hemolymph displacement in the abdominal hemocoel.

Furthermore, within the context of hemolymph circulation, the relative importance of abdominal contractions has been fiercely debated. After mathematically estimating the force of individual heart and abdominal contractions in lepidoteran and coleopteran pupa, Sláma concluded that the dorsal vessel is a weak organ incapable of propelling hemolymph against a pressure gradient, and postulated that the abdomen is the primary driver of hemolymph circulation [22], [28]. Kuusic and colleagues, on the other hand, countered that at low metabolic rates the dorsal vessel is sufficiently strong to initiate and maintain hemolymph flow and that it is only at higher metabolic rates that the abdomen is required to assist in pumping [23]. While none of the above studies (or ours) manipulated the manner in which either the heart or the abdomen contracts, and thus were unable to conclusively answer this question, our analyses suggest that the most important determinant of hemolymph circulation is the heart (our attempt to disrupt abdominal contractions while retaining normal heart function or vice versa using various anesthetic agents was unsuccessful). The heart contracts in both anterograde and retrograde directions with no periods of rest while the ventral abdomen contracts exclusively in the retrograde direction as series of consecutive contractions followed by periods of rest. Abdominal contraction periods are synchronized with the latter portion of anterograde heart periods but at all times it is the direction in which the heart contracts that determines the directional flow of hemolymph. We propose that, rather than functioning as the primary hemolymph pump, abdominal contractions allow the heart to extend the length and frequency of anterograde heart contraction periods. We hypothesize that during the course of anterograde heart periods the active shuttling of hemolymph from the abdomen to the thorax creates a pressure gradient between these two body regions. After this pressure gradient surpasses a particular threshold the translocation of hemolymph through the dorsal vessel becomes inefficient, requiring a heartbeat reversal to equilibrate hemocoel pressure. In adult mosquitoes, however, the heart contracts twice as often in the anterograde direction as it does in the retrograde direction [3], and we speculate that this is beneficial because of the higher efficiency of this pumping mechanism: anterograde heart contractions translocate hemolymph the entire length of the insect whereas retrograde heart contractions translocate hemolymph from the thoraco-abdominal junction to the posterior abdomen [3]. We hypothesize that by propelling extracardiac hemolymph in the retrograde direction, ventral abdominal contractions decrease the pressure differential between the thorax and the abdomen, thus allowing the dorsal vessel to extend anterograde heart contraction periods. For this reason, ventral abdominal contraction periods do not begin with the onset of anterograde heart periods, but instead after the pressure gradient has begun to decrease anterograde heart pumping efficiency.

Lastly, arguably the most debated purpose of abdominal contractions is potentiating active respiration, a function that would also be dependent on changes in hemolymph pressure resulting from asymmetrical circulation. Sláma and colleagues have shown that changes in hemolymph pressure lead to the compression and decompression of tracheoles, and that this is directly associated with mechanical outbursts of air [21], [22], [28]. However, using synchrotron X-ray imaging Westneat et al. showed in Coleoptera, Hymenoptera and Orthoptera that body movements and hemolymph circulation cannot by themselves account for the cyclic compression and expansion of tracheoles [31]. This notion is supported by Kuusic and colleagues, who report that not all insects coordinate respiration with heart or abdominal contractions, suggesting that a functional link between the circulatory and tracheal systems is not a universal feature of insects [20], [23], [32]. While the role of ventral abdominal contractions in insect respiration was not addressed in this study, we detected a conclusive link between abdominal contractions and heart contractions. This strong correlation, including the timing of the onset and end of abdominal contraction periods, suggests that at least in mosquitoes circulation is the primary function of these contractions and that tracheal ventilation could be a subsidiary outcome.

Mosquitoes are important pests and vectors of disease. Because of the global importance of mosquitoes, much research has focused on the molecular basis of susceptibility and resistance to insecticides and pathogens [33], [34], [35]. However, the physiological bases of these interactions have gone largely ignored. Most insecticides are absorbed through the cuticle before being disseminated with the hemolymph [36]. Similarly, most mosquito-borne pathogens enter the hemocoel and are subject to hemolymph currents during the course of their natural transmission cycles [15], [37]. The present study continues to expand our understanding of hemolymph circulation in mosquitoes, and provides insights into the forces that disseminate nutrients, hormones, and immune factors to all regions of the body, and the physiological interactions between pathogens and mosquitoes.

## Supporting Information

Figure S1 Effect of mosquito manipulation on heart contraction dynamics. Serial (paired) measurements of heart contraction dynamics were obtained from mosquitoes under control (neutral) and experimental conditions. Trials tested the effect of dorsal side-up positioning vs. lateral positioning (n = 13), no fluorescence illumination vs. fluorescence illumination (n = 8), low intensity incandescent light vs. high intensity incandescent light (n = 9), no injection vs. intrathoracic injection (n = 8), and 1 min recovery from cold-induced anesthesia vs. 5 min recovery from cold-induced anesthesia (n = 9). For all trials, measurements recorded were % time contracting in the anterograde (AG) direction (A), % time contracting in the retrograde (RG) direction (B), number of heartbeat reversals per minute (C), total contraction rate (D), anterograde contraction rate (E), and retrograde contraction rate (F). Overall, mosquito manipulation had no effect on contraction dynamics with the exception of fluorescence illumination, which affected 5 of the 6 factors measured (paired t-test or Wilcoxon matched pairs test). Comparison of the control (neutral) groups from each trial showed great biological variability but no significant difference between any of the factors measured (ANOVA). Median, center line; 50% of the data, box; 90% of the data, whiskers; mean, solid square.
(0.29 MB TIF)

Video S1 The ventral abdomen contracts in a retrograde direction during periods of anterograde heart contractions. Lateral view of the mosquito abdomen showing (1) 2 µm diameter fluorescent microspheres flowing through the heart (dorsal vessel) in anterograde and retrograde directions, and (2) periodic contractions of the ventral abdomen.
(3.26 MB MOV)

Video S2 Hemolymph flow in the lateral abdomen. Lateral view of the mosquito abdomen showing the flow of intrathoracically-injected fluorescent microspheres during the three principal contraction periods.
(2.67 MB MOV)

Video S3 Hemolymph flow in the ventral abdomen. Ventral view of the mosquito abdomen showing the flow of intrathoracically-injected fluorescent microspheres during the three principal contraction periods.
(2.30 MB MOV)

## References

[1] Klowden AJ (2007). Circulatory systems. Physiological systems in insects. 2nd ed.

[2] Nation JL (2008). Circulatory System. Insect physiology and biochemistry. 2nd ed.

[3] Glenn JD, King JG, Hillyer JF (2010). Structural mechanics of the mosquito heart and its function in bidirectional hemolymph transport.. J Exp Biol.

[4] Gerould J (1933). Orders of insects with heart-beat reversal.. The Biological Bulletin.

[5] Jones JC (1977). The circulatory system of insects.

[6] Wasserthal LT (1980). Oscillating haemolymph ‘circulation’ in the butterfly *Papilio machaon* L. revealed by contact thermography and photocell measurements.. J Comp Physiol.

[7] Wasserthal LT (2007). *Drosophila* flies combine periodic heartbeat reversal with a circulation in the anterior body mediated by a newly discovered anterior pair of ostial valves and ‘venous’ channels.. J Exp Biol.

[8] Dulcis D, Davis NT, Hildebrand JG (2001). Neuronal control of heart reversal in the hawkmoth *Manduca sexta*.. J Comp Physiol A.

[9] Duttlinger A, Mispelon M, Nichols R (2003). The structure of the FMRFamide receptor and activity of the cardioexcitatory neuropeptide are conserved in mosquito.. Neuropeptides.

[10] Ejaz A, Lange AB (2008). Peptidergic control of the heart of the stick insect, *Baculum extradentatum*.. Peptides.

[11] Nichols R (2006). FMRFamide-related peptides and serotonin regulate *Drosophila melanogaster* heart rate: mechanisms and structure requirements.. Peptides.

[12] Dulcis D, Levine RB (2005). Glutamatergic innervation of the heart initiates retrograde contractions in adult *Drosophila melanogaster*.. J Neurosci.

[13] Dulcis D, Levine RB, Ewer J (2005). Role of the neuropeptide CCAP in *Drosophila* cardiac function.. J Neurobiol.

[14] Pass G (2000). Accessory pulsatile organs: evolutionary innovations in insects.. Annu Rev Entomol.

[15] Hillyer JF, Barreau C, Vernick KD (2007). Efficiency of salivary gland invasion by malaria sporozoites is controlled by rapid sporozoite destruction in the mosquito haemocoel.. Int J Parasitol.

[16] Hillyer JF, Schmidt SL, Christensen BM (2003). Rapid phagocytosis and melanization of bacteria and *Plasmodium* sporozoites by hemocytes of the mosquito *Aedes aegypti*.. J Parasitol.

[17] Hillyer JF, Schmidt SL, Christensen BM (2003). Hemocyte-mediated phagocytosis and melanization in the mosquito *Armigeres subalbatus* following immune challenge by bacteria.. Cell Tissue Res.

[18] Gillett JD (1982). Circulatory and ventilatory movements of the abdomen in mosquitoes.. Proc R Soc Lond B.

[19] Koladich PM, Tobe SS, McNeil JN (2002). Enhanced haemolymph circulation by insect ventral nerve cord: hormonal control by *Pseudaletia unipuncta* allatotropin and serotonin.. J Exp Biol.

[20] Kuusik A, Harak M, Hiiesaar K, Metspalu L, Tartes U (1996). Different types of external gas exchange found in pupae of greater wax moth *Galleria mellonella* (Lepidoptera:Pyralidae).. Eur J Entomol.

[21] Slama K, Neven L (2001). Active regulation of respiration and circulation in pupae of the codling moth (*Cydia pomonella*).. J Insect Physiol.

[22] Slama K (2008). Extracardiac haemocoelic pulsations and the autonomic neuroendocrine system (coelopulse) of terrestrial insects.. Terrestrial Arthropod Reviews.

[23] Tartes U, Vanatoa A, Kuusik A (2002). The insect abdomen–a heartbeat manager in insects?. Comp Biochem Physiol, Part A Mol Integr Physiol.

[24] Slama K (1984). Recording of haemolymph pressure pulsations from the insect body surface.. J Comp Physiol B.

[25] Slama K, Baudry-Partiaoglou N, Provansal-Baudez A (1979). Control of extracardiac haemolymph pressure pulses in *Tenebrio molitor*.. J Insect Physiol.

[26] Slama K (1976). Insect hemolymph pressure and its determination.. Acta Entomol Bohemos.

[27] Heinrich B (1976). Heat exchange in relation to blood flow between thorax and abdomen in bumblebees.. J Exp Biol.

[28] Slama K (1999). Active regulation of insect respiration.. Ann Entomol Soc Am.

[29] Wasserthal LT (1976). Heartbeat reversal and its coordination with accessory pulsatile organs and abdominal movements in Lepidoptera.. Experientia.

[30] Ichikawa T (2008). Periodic abdominal pumping supports leg development during metamorphosis in tenebrionid beetle *Zophobas atratus*.. Comp Biochem Physiol, Part A Mol Integr Physiol.

[31] Westneat MW, Betz O, Blob RW, Fezzaa K, Cooper WJ (2003). Tracheal respiration in insects visualized with synchrotron x-ray imaging.. Science.

[32] Kuusik A, Martin A, Mänd M (2004). Cyclic release of carbon dioxide accompanied by abdominal telescoping movements in forager ants of *Formica polyctena* (Hymenoptera, Formicidae).. Physiological Entomology.

[33] Aliota MT, Fuchs JF, Rocheleau TA, Clark AK, Hillyer JF (2010). Mosquito transcriptome profiles and filarial worm susceptibility in *Armigeres subalbatus*.. PLoS Negl Trop Dis.

[34] Baton L, Robertson A, Warr E, Strand M, Dimopoulos G (2009). Genome-wide transcriptomic profiling of *Anopheles gambiae* hemocytes reveals pathogen-specific signatures upon bacterial challenge and *Plasmodium berghei* infection.. BMC Genomics.

[35] Hemingway J, Hawkes NJ, McCarroll L, Ranson H (2004). The molecular basis of insecticide resistance in mosquitoes.. Insect Biochem Mol Biol.

[36] Pedigo LP (2002). Conventional insecticides for management. Entomology and pest management. 4th ed.

[37] Hillyer JF, Söderhäll K (2010). Mosquito immunity.. Invertebrate Immunity: Landes BioScience.

