# Supplementary figures and images for: Contraction of the Ventral Abdomen Potentiates Extracardiac Retrograde Hemolymph Propulsion in the Mosquito Hemocoel
Source: PLoS One. 2010 Sep 23;5(9):e12943. doi: 10.1371/journal.pone.0012943 (PMC2944847; doi:10.1371/journal.pone.0012943)

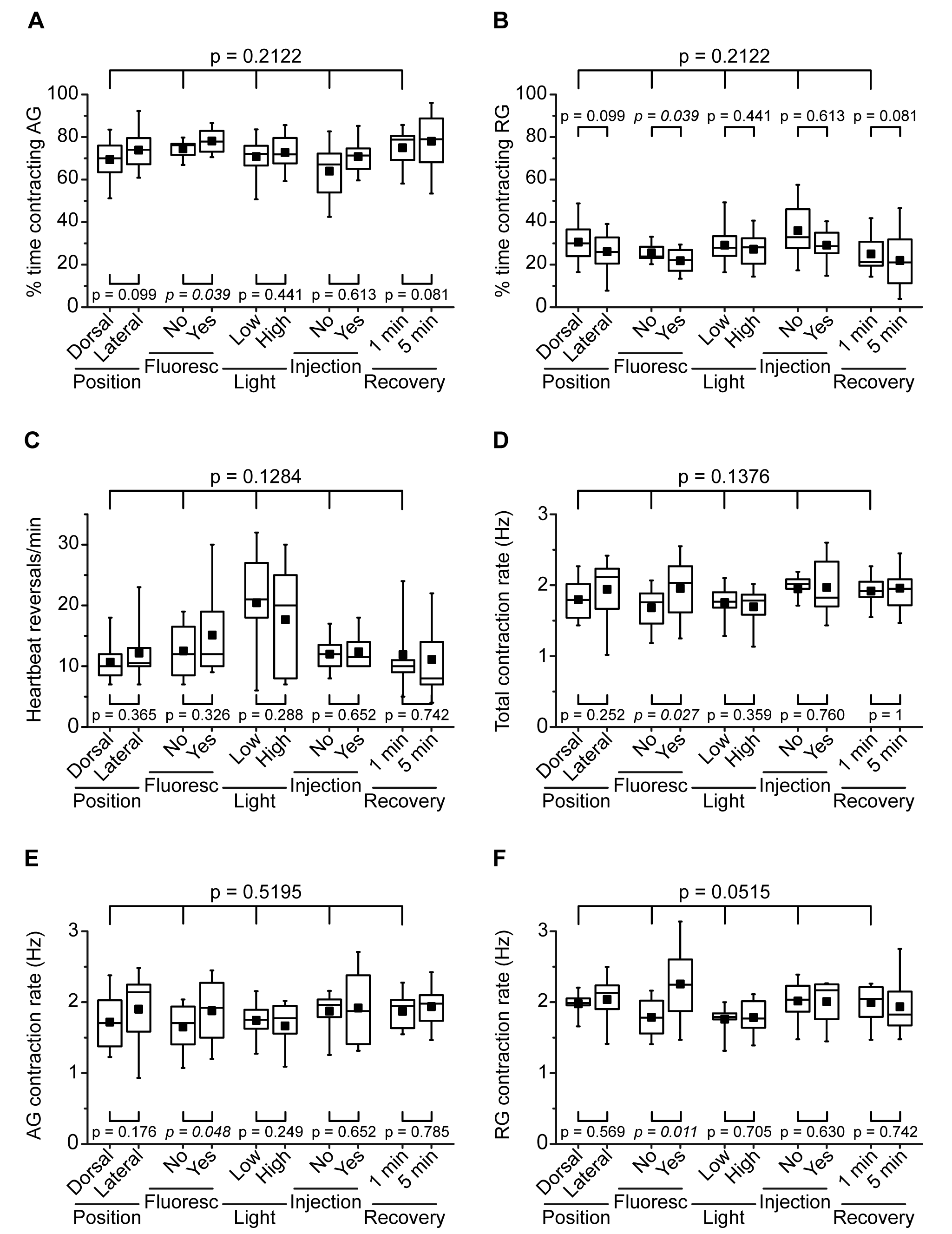

Supplement: Figure S1 — Effect of mosquito manipulation on heart contraction dynamics. Serial (paired) measurements of heart contraction dynamics were obtained from mosquitoes under control (neutral) and experimental conditions. Trials tested the effect of dorsal side-up positioning vs. lateral positioning (n = 13), no fluorescence illumination vs. fluorescence illumination (n = 8), low intensity incandescent light vs. high intensity incandescent light (n = 9), no injection vs. intrathoracic injection (n = 8), and 1 min recovery from cold-induced anesthesia vs. 5 min recovery from cold-induced anesthesia (n = 9). For all trials, measurements recorded were % time contracting in the anterograde (AG) direction (A), % time contracting in the retrograde (RG) direction (B), number of heartbeat reversals per minute (C), total contraction rate (D), anterograde contraction rate (E), and retrograde contraction rate (F). Overall, mosquito manipulation had no effect on contraction dynamics with the exception of fluorescence illumination, which affected 5 of the 6 factors measured (paired t-test or Wilcoxon matched pairs test). Comparison of the control (neutral) groups from each trial showed great biological variability but no significant difference between any of the factors measured (ANOVA). Median, center line; 50% of the data, box; 90% of the data, whiskers; mean, solid square. (0.29 MB TIF) [file pone.0012943.s001.tif]
